# Identification and validation of two major QTLs for spikelet number per spike in wheat (*Triticum aestivum* L.)

**DOI:** 10.3389/fpls.2023.1144486

**Published:** 2023-05-10

**Authors:** Xiaoyu Yi, Yingtong Ye, Jinhui Wang, Zhen Li, Jiamin Li, Yuqi Chen, Guoyue Chen, Jian Ma, Zhien Pu, Yuanying Peng, Pengfei Qi, Yaxi Liu, Qiantao Jiang, Jirui Wang, Yuming Wei, Youliang Zheng, Wei Li

**Affiliations:** ^1^ State Key Laboratory of Crop Gene Exploration and Utilization in Southwest China, Sichuan Agricultural University, Chengdu, China; ^2^ College of Agronomy, Sichuan Agricultural University, Chengdu, China; ^3^ Triticeae Research Institute, Sichuan Agricultural University, Chengdu, China

**Keywords:** genetic linkage map, multiple spikelets, QTL mapping, exon capture trapping, genetic localization

## Abstract

The total number of spikelets (TSPN) and the number of fertile spikelets (FSPN) affect the final number of grains per spikelet in wheat. This study constructed a high-density genetic map using 55K single nucleotide polymorphism (SNP) arrays from a population of 152 recombinant inbred lines (RIL) from crossing the wheat accessions 10-A and B39. Twenty-four quantitative trait loci (QTLs) for TSPN and 18 QTLs for FSPN were localized based on the phenotype in 10 environments in 2019–2021. Two major QTLs, *QTSPN/QFSPN.sicau-2D.4* (34.43–47.43 Mb) and *QTSPN/QFSPN.sicau-2D.5*(32.97–34.43 Mb), explained 13.97%–45.90% of phenotypic variation. Linked kompetitive allele-specific PCR (KASP) markers further validated these two QTLs and revealed that *QTSPN.sicau-2D.4* had less effect on TSPN than *QTSPN.sicau-2D.5* in 10-A×BE89 (134 RILs) and 10-A×Chuannong 16 (192 RILs) populations, and one population of Sichuan wheat (233 accessions). The alleles combination haplotype 3 with the allele from 10-A of *QTSPN/QFSPN.sicau-2D.5* and the allele from B39 of *QTSPN.sicau-2D.4* resulted in the highest number of spikelets. In contrast, the allele from B39 for both loci resulted in the lowest number of spikelets. Using bulk-segregant analysis–exon capture sequencing, six SNP hot spots that included 31 candidate genes were identified in the two QTLs. We identified *Ppd-D1a* from B39 and *Ppd-D1d* from 10-A and further analyzed *Ppd-D1* variation in wheat. These results identified loci and molecular markers with potential utility for wheat breeding and laid a foundation for further fine mapping and cloning of the two loci.

## 1 Introduction

Common wheat (*Triticum aestivum* L., 2n = 6x = 42, AABBDD) is a hexaploid species (Consortium et al., 2014) with a large and complex genome (Bonjean and Angus, 2001; Choulet et al., 2014). Wheat is the most widely grown, with the world’s highest production and most traded food crop, sustaining one-third of the world’s population (Gupta et al., 2008). Wheat yield is a complex trait affected by multiple genetic and environmental factors. Usually, it comprises three main components: spike number per unit area, grain number per spike, and thousand-grain weight (Kuzay et al., 2019). Among these components, grain number per spike is predominantly determined by the total number of spikelets and the number of fertile spikelets, which show low environmental sensitivity and high heritability (Zhang et al., 2018; Gao et al., 2019). Therefore, understanding the genetic factors that regulate spikelet number is crucial for improving wheat yield.

The wheat spikes of wheat are characteristically unbranched inflorescences comprising an indeterminate number of florets (Koppolu and Schnurbusch, 2019). Light and temperature are the dominant factors impacting wheat spike development (Langer and Hanif, 1973). Spike fertility is affected by environmental and genetic factors (Guo et al., 2015; Sakuma et al., 2019) and is a complex quantitative trait controlled by multiple genes (Ma et al., 2007). The number, chromosomal location, and genetic effects of these multiple genes must be determined to generate optimal genotypes in breeding.

Only a few genes associated with spikelet numbers have been cloned to date. For example, *WAPO1* on chromosome 7AL regulates spikelet number by affecting inflorescence development (Kuzay et al., 2019). The domestication gene *Q* on chromosome 5A regulates spikelet density (Simons et al., 2006; Zhang et al., 2011; Debernardi et al., 2017; Greenwood et al., 2017). The genes *TaDEP1* (homologous to rice *DENSE AND ERECT PANICLE 1*) (Vavilova et al., 2019; Huang et al., 2022) and *TaCOL-b5* modify wheat spike structure to increase yield (Zhang et al., 2022). The genes mined on chromosome 2D include the photoperiod-sensitive gene *Ppd-D1*, *TaMOC1* (the ortholog of rice *MONOCULM 1*), and the *FZP* gene. The *Ppd* system includes the *Ppd-A1*, *Ppd-B1*, and *Ppd-D1* genes located in the second homologous group, which are responsive to changes in photoperiod (i.e., day length) (Law et al., 1978; Scarth and Law, 1983). The *FZP* gene is a crucial regulator of inflorescence development and a determinant of complex spikelet formation (Dobrovolskaya et al., 2015).

Many quantitative trait loci (QTLs) for spikelet number have been identified on almost all the 21 chromosomes of wheat. The QTLs associated with spikelet number explain 2.15%–67.6% of the phenotypic variation of spikelets (Cui et al., 2012; Dan et al., 2017). Many QTLs related to spikelet number are located on chromosome 2D, and their physical locations range from 9.34 to 648.11 Mb on this chromosome (Li et al., 2002; Sourdille et al., 2003; Quarrie et al., 2006; Ma et al., 2007; Cui et al., 2012; Liu et al., 2014; Zhai et al., 2016; Deng et al., 2019; Ma et al., 2019; Li et al., 2021; Saini et al., 2022). However, few of these QTLs are stably correlated with spikelet numbers in different genetic backgrounds and environments. The contribution rate is relatively low, hinders these QTLs’ application in wheat breeding.

This study constructed a genetically stable high-generation (F_2:8_) recombinant inbred line (RIL) population using the wheat germplasm resources 10-A and B39, which differ significantly in spikelet number. The interactions between the mapped QTLs were analyzed. Exon capture sequencing was used to analyze the differential single nucleotide polymorphism (SNP) loci within the main QTL interval to detect hot spots and predict the target gene by combining of an expression database and differential SNP loci. The reported genes located in the interval were cloned and identified. The results identify loci and molecular markers of potential utility for wheat breeding and lay a foundation for further fine mapping and cloning of the loci.

## 2 Materials and methods

### 2.1 Plant materials

The wheat germplasm 10-A is an unbranched common multi-spikelet line formed by introducing of the heterologous genes of rye, which has the characteristics of multi-flower and multi-grain and large spikes. The wheat lines B39 and Chuannong 16 (CN16) were bred by the Triticeae Research Institute of Sichuan Agricultural University, China. BE89 is descended from the cross between ‘Batavia’ and ‘Erine’.

Four populations were used in this study. An F_2:8_ RIL (152 lines) population derived from the cross of 10-A and B39 was used for the construction of genetic maps and mapping QTLs. The remaining three populations comprised a population of 134 F2:8 RIL lines derived from the cross between 10-A and BE89, a population of 192 F_2:5_ RIL lines from the cross of 10-A and CN16, and a natural population (CD) comprising 233 accessions of Sichuan landraces and cultivars (**Supplementary Tables 3**, **4**). The Triticeae Research Institute of Sichuan Agricultural University provided the materials.

### 2.2 Phenotyping and statistical analysis

Phenotypic traits of the 10-A/B39 population were measured in 10 environments: Wenjiang in 2019, 2020, and 2021 (E1, E4, and E7), Chongzhou in 2019, 2020, and 2021 (E2, E5, and E8), Ya’an in 2019, 2020, and 2021 (E3, E6, and E9), and Shifang in 2021 (E10). The three validation populations were planted at the Wenjiang Experimental Teaching Base of Sichuan Agricultural University in 2021. Two replications were performed in each environment for each population. The local practices for wheat production performed field management and disease control. At maturity, five representative plants of similar growth status were randomly selected to measure agronomic traits for the main spike, comprising the heading period, flowering period, productive tillers number (FTN), plant height (PH), spike length (SL), awn length (AL), TSPN, FSPN, number of sterile spikelets per spike (SSPN), grain number per spike (GNS), and thousand-kernel weight (TKW). SL was measured as the distance from the rachis’s base to the terminal spikelet’s tip, excluding the awns. The spikelet number per spike was determined by counting the number of spikelets in the main spike.

### 2.3 Linkage map construction and QTL detection

The QTL mapping analysis was performed using the 152 RILs of the 10-A/B39 population. Leaves were sampled at the seedling stage and extracted DNA using the cetyltrimethylammonium bromide (CTAB) method. The ratio of *A_260_/A_280_* of the DNA extracts was 1.70/2.10, the ratio of *A_260_/A_230_* was >1.50, and the DNA concentration was >25 ng/μL. The Wheat_55K SNP chip was used for SNP sequencing and genotyping of the two parental and RIL populations (predominantly performed by the China Golden Marker (Beijing) Biotechnology Co., Ltd., Beijing, China). Mapping was conducted using the inclusive composite interval mapping function of QTL IciMapping 4.1 (https://www.isbreeding.net) software. The environment-specific QTLs were detected using the bi-parental population’s module with walking step = 0.001 cM, probability value for entering variables in stepwise regression of phenotype on marker variables (PIN) = 0.0001, and the logarithm of the odds (LOD) score ≥ 3. A QTL that explained more than 10% of the phenotypic variation and detected in more than four environments (including the best linear unbiased prediction [BLUP] dataset) was treated as a major stable QTL. The QTL loci were named according to the International Rules of Genetic Nomenclature: sicau indicates Sichuan Agricultural University, TSPN stands for total spikelet number, and FSPN represents the number of effective spikelets.

### 2.4 Marker development and QTL validation in different genetic backgrounds

Based on the preliminary QTL mapping results, the lateral markers of the main QTLs were converted into kompetitive allele-specific PCR (KASP) markers to track the main QTLs, and new markers were developed within the interval. The flanking markers of the major QTLs were converted into KASP primers by Li et al. (2021). The addition of a FAM signal and a HEX signal to the primers differentiated the two parental genotypes. The KASP assay results were detected with the Bio-Rad CFX96 Real-Time PCR system. The newly developed markers were integrated into the genetic map to determine whether the developed KASP markers were tightly linked to major QTLs,. QTL IciMapping 4.2 software targeted the re-positioned QTLs in the 10-A/B39 population of 152 RILs.

### 2.5 Exon capture sequencing

In the F_2:10_ RIL 10-A/B39 population, 20 lines with the maximum TSPN and 20 with the minimum TSPN were selected to generate extreme pools. Leaves were sampled at the seedling stage, and DNA was extracted using the CTAB method. The respective DNA extracts were mixed to form the maximum and minimum extreme pools. The same method was used to extract DNA from 10-A and B39 to construct mixed parental pools. Sequencing and genotyping were predominantly performed by the Chengdu Tiancheng Future Technology Co., Ltd., Chengdu, China). The QTLseqr software package was used to calculate the allele frequency within the segment based on the SNP index and G′ value of the sliding window, as well as the allele frequency difference for the two pools, to detect the segment with the most strongly significant difference.

### 2.6 Candidate gene prediction

The interval range was further narrowed within the target QTL interval based on the marker sites of the SNP differences determined by exon capture sequencing. The Ensembl Plants database (http://plants.ensembl.org/index.html), in combination with wheat IWGSC RefSeq v1.1 gene annotations, was used to compare the physical location of the variant site and to locate the mutation site. WheatOmics 1.0 (http://202.194.139.32/) was used to analyze the gene expression levels in the different tissue sites. The target genes were annotated with gene ontology (GO) terms using the GOEnrichment tool in the TriticaeGeneTribe database (http://wheat.cau.edu.cn/TGT/index.html).

### 2.7 Gene cloning

The CTAB method was used to extract DNA for the 10-A and B39 parents and offspring. Specific primers were designed using NCBI (https://www.ncbi.nlm.nih.gov/) based on the genome sequence (DQ885766) of the Chinese spring light cycle gene *Ppd-D1*. The PCR amplifications were conducted using Phanta Max Super-Fidelity DNA Polymerase (1 U/μL, Vazyme Biotech Co., Ltd) following the manufacturer’s instructions. Amplification of *Ppd-D1* was performed with the gene-specific primers for the parental materials and ‘Chinese Spring’. The PCR products were electrophoresed in 2% agarose gel (160 V, 400 mA, 25 min) and observed with a gel imager (Tocan 240). The target bands were excised and placed in a 2.0 mL EP tube. The PCR products were recovered using the OMEGA Best Standard Agarose gel DNA recovery kit following the manufacturer’s instructions. The PCR products were connected with the target gene cloning vector (T-vector) using the Novizan 5min™ TA/Blunt-Zero Cloning Kit. The chemically competent *Escherichia coli* strain DH5α (Trelief™ 5α Chemically Competent Cell) cells were transformed by heat shock. The monoclonals were detected by PCR amplification. The monoclonals were cultured on an LB medium, and the universal primer M13 was used for PCR amplification of the insert in the cloning vector. Amplification of the target gene sequence indicated that the monoclonal was positive. The bacterial suspension containing the target band was submitted to the Beijing Qingke Biotechnology Co., Ltd. (Beijing, China) for sequencing. The *Ppd-D1* gene sequence was analyzed using DNAMAN 9.0 (https://www.lynnon.com/qa.html) software.

## 3 Results

### 3.1 Phenotypic variation

**Figure 1:**
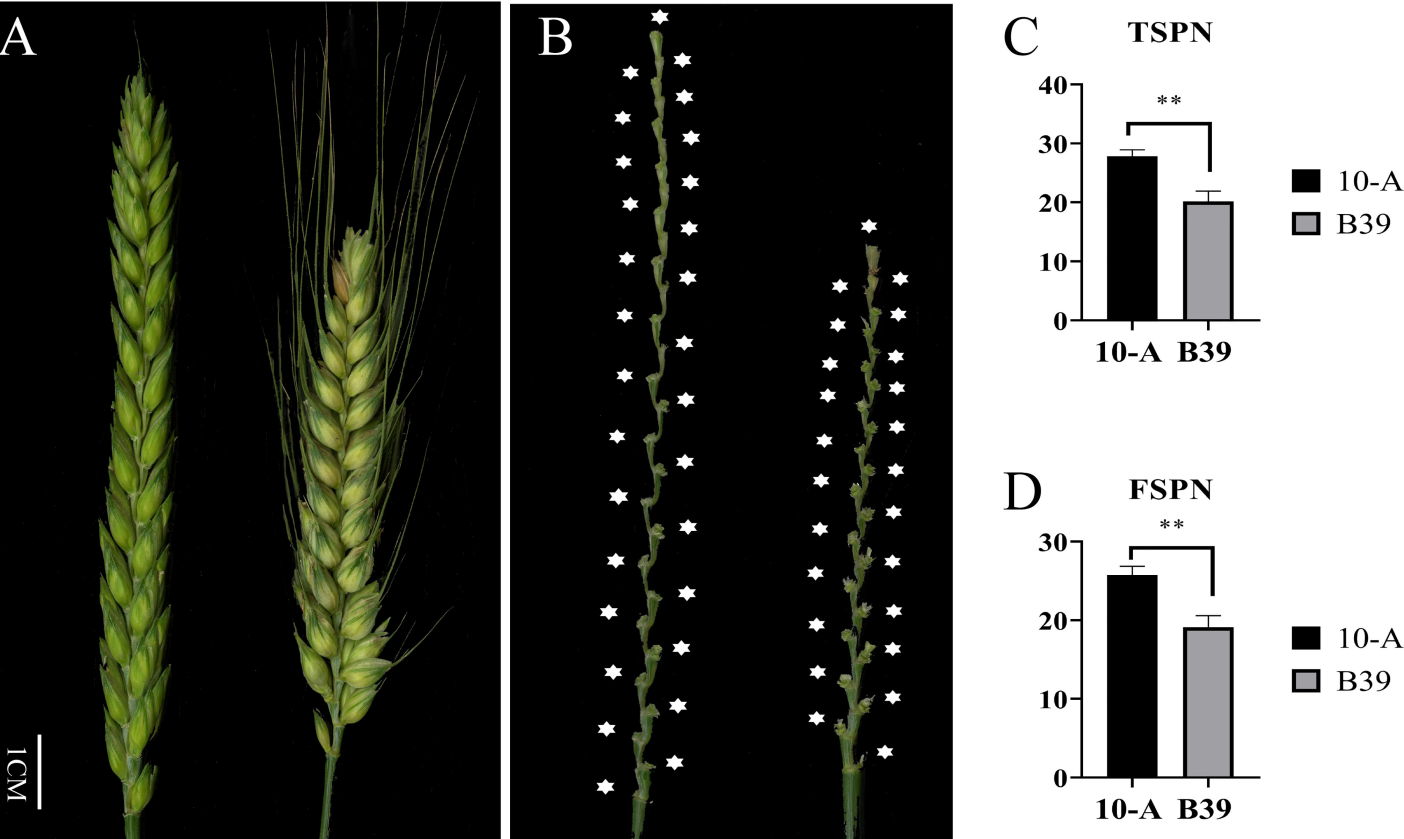
Spike morphology of the parents. The morphology of spikes of 10-A and B39 at the filling period **(A)** and rachis **(B)**. The mean of total spikelet number per spike (TSPN) **(C)** and the fertile spikelet number per spike (FSPN) **(D)** of 10-A and B39 at maturity in ten environments. ** represents significance at P < 0.01.

In the ten environments (**Figure 1**), the TSPN of 10-A ranged from 25 to 30, and the average was 27.87. The FSPN of 10-A ranged from 23 to 29, averaging 25.76. The TSPN of B39 ranged from 16 to 24; the average was 20.17. The number of effective spikelets of B39 varied from 15 to 23, and the average was 19.13. Comparing TSPN and FSPN between 10-A and B39 in all environments, the target traits of the female parent (10-A) were significantly higher than those of the male parent (B39).

Among the ten environments and the BLUP dataset (**Supplementary Table 1**), the number of spikelets in the population of 152 RILs derived from 10-A and B39 ranged from 14 to 38. The mean value in the population ranged from 22.03 to 26.44, and the coefficient of variation ranged from 8.14% to 14.04% fluctuated, and the same traits did not fluctuate much in different environments. The broad-sense heritability was 0.84 for TSPN and 0.76 for FSPN, indicating that genetic factors mainly controlled the traits.

### 3.2 Correlation analysis

Correlation analysis between TSPN and FSPN in all environments and BLUP values showed that TSPN and FSPN were highly significantly positively correlated in all environments (**Supplementary Figure 1**). In the 10-A/B39 population, significant positive correlations between TSPN and FSPN (**Supplementary Table 2**), and negative correlations between TSPN and TKW, were observed. TSPN was strongly positively correlated with SL, SSPN, heading period, and flowering period and weakly positively correlated with TGW. No significant correlation was detected for TSPN with FTN, PH, and AL. Significant correlations were detected for FSPN with SL, heading period, and flowering periods.

### 3.3 Comparison of genetic maps and physical maps

The scores for the probes were classified into six categories by the Affymetrix software: (I) Poly High Resolution (PHR) (26,485; 49.9%); (II) No Minor Homozygote (10457; 19.7%); (III) Off-Target Variant (3437; 6.5%); (IV) Mono High Resolution (278; 0.5%); (V) Call Rate Below Threshold (1475; 2.8%); and (VI) Other (10931; 20.6%). Only the probes from the first group (PHR) with the highest reliability were retained (defined as frequency < 0.3). Ultimately, 8719 SNP markers were used for linkage analysis and map construction. Linkage analysis revealed that 1354 bin markers were mapped on the genetic maps. The total genetic distance of the genetic linkage map was 2407.17 cM, the average genetic distance was 2.42 cM/SNP, and the map contained 1247 SNP markers (**Table 1**). The percentages of markers contained in the chromosomes of subgenomes A, B, and D were 38.89%, 37.77%, and 23.34%, respectively. The SNPs were distributed on all 21 chromosomes. Based on the SNP flanking sequences, we assigned all mapped SNPs to the wheat genome assembly IWGSC RefSeq v1.0. The SNP order in the present genetic map showed good agreement with that in the wheat genome assembly (**Supplementary Figure 2**).

**Table 1:** The information of genetic map.

Chromosome	The original markernumberr	Effective markernumber	Length (cM)	Average (cM/SNP)
1A (1)	51	42	82.3	1.96
1A (2)		7	23.63	3.38
1B	87	86	101.68	1.18
1D	28	21	73.91	3.52
2A	49	48	107.04	2.23
2B	81	81	106.95	1.32
2D (1)	34	22	48.49	2.2
2D (2)		10	12.07	1.21
3A	101	99	180.66	1.82
3B	74	74	126.73	1.71
3D (1)	44	22	90.41	4.11
3D (2)		8	61.57	7.7
3D (3)		7	27.03	3.86
4A	76	75	142.32	1.9
4B	61	61	105.67	1.73
4D	35	34	80.5	2.37
5A	82	82	114.42	1.4
5B	69	67	128.79	1.92
5D	62	46	148.88	3.24
6A	43	39	61.86	1.59
6B	37	36	96.33	2.68
6D	50	46	147.4	3.2
7A	94	93	161.14	1.73
7B	66	66	69.46	1.05
7D	81	75	107.95	1.44
A	496	485	873.36	2
B	475	471	735.61	1.66
D	334	291	798.2	3.28
SUM	1305	1247	2407.17	2.42

### 3.4 Preliminary mapping of QTLs for TSPN and FSPN

Mapping of QTLs was performed based on the phenotypic data and BLUP values for TSPN in the ten environments (**Tables 1**, **2**). Twenty-four QTLs for TSPN were identified and distributed on chromosomes 1B (two QTLs), 2A (three), 2D (three), 3A (one), 3B (one), 3D (one), 4B (five), 5A (four), 5B (one), and 7D (three). The proportion of the phenotypic variance explained ranged from 3.36% to 20.92%. *QTSPN.sicau-2D.1* was detected between *AX-109465277* and *AX-109785183* in ten environments and the BLUP dataset and explained 10.38%–20.92% of the total phenotypic variation with LOD values of 4.10–13.10. *QTSPN.sicau-2D.1* was repeatedly detected in the ten environments and BLUP dataset. This QTL was considered an environmentally stable and major QTL, and its additive effect was derived from 10-A. *QTSPN.sicau-2D.3* was detected in six environments (E2, E3, E4, E5, E6, and BLUP), and explained 12.59%–17.52% of the total phenotypic variance with LOD values of 4.77–10.89. *QTSPN.sicau-2A.3* was detected in three environments (E2, E4, and BLUP) and explained 6.57%–10.11% of the phenotypic variance. Both *QTSPN.sicau-2D.2* and *QTSPN.sicau-3B.1* were detected in two environments, while the remaining QTLs were detected in a single environment, explaining 3.36%–11.34% of the phenotypic variance. Among these QTLs, *QTSPN.sicau-3B.1*, *QTSPN.sicau-5B.1*, *QTSPN.sicau-7D.1*, *QTSPN.sicau-7D.2*, and *QTSPN.sicau-7D.3* alleles were derived from B39. The remaining QTL alleles were derived from 10-A.

**Table 2:** QTL mapping for the total spikelets per spike and the fertile spikelets per spike in wheat from “10-A×B39”RIL population at 55K SNP chip.

Trait	QTLs	Environment	Chromosome	Position (cM)	LeftMarker	RightMarker	LOD	PVE(%)	Add
TSPN	*QTSPN.sicau-1B.1*	E1	1B	20	*AX-108895682*	*AX-111287959*	2.7189	6.3437	0.6068
	*QTSPN.sicau-1B.2*	E6	1B	12	*AX-108928321*	*AX-110675553*	5.2687	7.1914	0.8614
	*QTSPN.sicau-2A.1*	E6	2A	72	*AX-108856880*	*AX-110126474*	4.5695	6.4432	0.8259
	*QTSPN.sicau-2A.2*	E9	2A	73	*AX-110126474*	*AX-111140902*	3.4572	7.7238	0.8914
	*QTSPN.sicau-2A.3*	E2	2A	77	*AX-111464687*	*AX-110512107*	3.1879	6.5794	0.6131
		E4	2A	77			4.9272	10.1126	0.9497
		BLUP	2A	77			5.2609	7.8744	0.6143
	*QTSPN.sicau-2D.1*	E1	2D	0	*AX-109785183*	*AX-109465277*	6.5382	15.7558	0.9861
		E2	2D	0			7.3304	15.8397	0.9652
		E3	2D	0			6.9189	18.8964	1.1766
		E4	2D	0			9.6504	20.9201	1.386
		E5	2D	0			9.3524	15.8207	1.3766
		E6	2D	0			13.0977	19.4852	1.461
		E7	2D	1			4.8517	10.3833	0.7461
		E8	2D	0			4.0956	11.4401	0.8116
		E9	2D	0			6.2099	13.9613	1.2178
		E10	2D	0			4.102	11.1578	0.6289
		BLUP	2D	0			12.2369	19.8726	0.9905
	*QTSPN.sicau-2D.2*	E1	2D	4	*AX-109842248*	*AX-111589614*	4.4465	10.5323	0.8019
		E8	2D	4			2.8715	8.1705	0.6807
	*QTSPN.sicau-2D.3*	E2	2D	2	*AX-89728114*	*AX-109842248*	7.2589	15.8518	0.9577
		E3	2D	0			4.7708	12.5997	0.9516
		E4	2D	1			7.0872	15.2526	1.1748
		E5	2D	0			9.677	16.455	1.3905
		E6	2D	1			10.2669	14.9818	1.2692
		BLUP	2D	2			10.8968	17.5225	0.9245
	*QTSPN.sicau-3A.1*	E7	3A	79	*AX-86165946*	*AX-109621355*	2.9281	5.6248	0.5352
	*QTSPN.sicau-3B.1*	E5	3B	75	*AX-108880481*	*AX-111564046*	5.8006	9.5945	-1.0422
		E7	3B	75			3.0817	5.8924	-0.5495
	*QTSPN.sicau-3D.1*	E7	3D	12	*AX-89577616*	*AX-109859243*	2.7095	5.2169	0.5193
	*QTSPN.sicau-4B.1*	E9	4B	44	*AX-109827483*	*AX-110361956*	2.9148	6.2412	0.7921
	*QTSPN.sicau-4B.2*	E8	4B	53	*AX-109948933*	*AX-108929144*	2.9118	8.0111	0.659
	*QTSPN.sicau-4B.3*	E5	4B	48	*AX-110382985*	*AX-111719562*	2.832	4.4575	0.7118
	*QTSPN.sicau-4B.4*	E10	4B	30	*AX-111162353*	*AX-108820969*	4.0788	11.2478	0.6191
	*QTSPN.sicau-4B.5*	E2	4B	17	*AX-89469514*	*AX-89554870*	3.3764	6.9524	0.6192
	*QTSPN.sicau-5A.1*	E1	5A	61	*AX-109974768*	*AX-109004929*	2.8795	6.6348	0.6218
	*QTSPN.sicau-5A.2*	E5	5A	97	*AX-110472228*	*AX-109432156*	3.1514	4.8581	0.7388
	*QTSPN.sicau-5A.3*	E6	5A	88	*AX-111539014*	*AX-109346674*	2.6211	3.3654	0.5907
	*QTSPN.sicau-5A.4*	BLUP	5A	91	*AX-111463028*	*AX-109504449*	4.0823	5.8567	0.5222
	*QTSPN.sicau-5B.1*	E6	5B	95	*AX-108950987*	*AX-109518159*	3.6868	4.7768	-0.7045
	*QTSPN.sicau-7D.1*	E7	7D	44	*AX-109030892*	*AX-111729400*	3.03	5.794	-0.5514
	*QTSPN.sicau-7D.2*	E10	7D	45	*AX-109194960*	*AX-111435770*	2.9372	8.0622	-0.5295
	*QTSPN.sicau-7D.3*	BLUP	7D	106	*AX-110502471*	*AX-111932357*	2.5849	3.6134	-0.4095
FSPN	*QFSPN.sicau-1B.1*	E6	1B	9	*AX-110409346*	*AX-109563613*	4.5127	7.013	0.9279
	*QFSPN.sicau-2A.1*	E4	2A	76	*AX-109517079*	*AX-108803612*	5.0366	9.5796	0.8678
	*QFSPN.sicau-2A.2*	E6	2A	73	*AX-110126474*	*AX-111140902*	5.261	8.4451	1.0299
	*QFSPN.sicau-2A.3*	E2	2A	77	*AX-111464687*	*AX-110512107*	3.0892	7.552	0.6528
		E8	2A	77			3.4476	8.5095	0.7128
		E9	2A	77			3.3871	4.0976	0.8238
		BLUP	2A	77			5.2162	8.9481	0.5482
	*QFSPN.sicau-2D.1*	E1	2D	0	*AX-109785183*	*AX-109465277*	4.392	15.3243	0.8975
		E2	2D	0			4.6134	11.2551	0.8088
		E3	2D	0			5.9734	17.0008	1.1391
		E4	2D	0			9.437	18.8335	1.2257
		E5	2D	0			3.2351	8.5944	0.816
		E6	2D	0			10.8929	18.5046	1.5513
		E7	2D	1			5.0528	14.3942	0.8445
		E8	2D	0			4.8116	11.9722	0.8581
		E9	2D	0			5.576	6.8096	1.0772
		BLUP	2D	0			11.6815	19.8385	0.8288
	*QFSPN.sicau-2D.2*	E8	2D	4	*AX-109842248*	*AX-111589614*	3.1075	7.7881	0.6871
	*QFSPN.sicau-2D.3*	E2	2D	1	*AX-89728114*	*AX-109842248*	6.1488	15.7279	0.9478
		E3	2D	0			3.3399	9.1248	0.8265
		E5	2D	0			5.1711	14.1553	1.0373
		E6	2D	0			7.9537	12.8947	1.2827
		E7	2D	1			2.9891	7.7744	0.6192
		BLUP	2D	1			8.9419	14.8558	0.7119
	*QFSPN.sicau-3B.1*	E5	3B	75	*AX-108880481*	*AX-111564046*	2.9999	8.1053	-0.7703
	*QFSPN.sicau-3D.1*	E7	3D	12	*AX-89577616*	*AX-109859243*	2.5808	6.4628	0.5558
	*QFSPN.sicau-3D.2*	E4	3D	24	*AX-111408918*	*AX-111366210*	3.0756	5.71	-0.6826
	*QFSPN.sicau-4B.1*	E9	4B	44	*AX-109827483*	*AX-110361956*	13.1178	18.276	1.7164
	*QFSPN.sicau-4B.2*	E9	4B	49	*AX-109931786*	*AX-110476859*	6.5632	8.1848	-1.1485
	*QFSPN.sicau-5A.1*	E4	5A	99	*AX-109432156*	*AX-111026217*	3.0757	5.5765	0.6465
	*QFSPN.sicau-5A.2*	E6	5A	85	*AX-111756872*	*AX-108861269*	3.2251	4.8856	0.7832
	*QFSPN.sicau-5A.3*	BLUP	5A	91	*AX-111463028*	*AX-109504449*	4.1688	6.3233	0.4545
	*QFSPN.sicau-6D.1*	E9	6D	19	*AX-108954528*	*AX-89511305*	2.5833	3.1246	0.7152
	*QFSPN.sicau-7B.1*	E3	7B	52	*AX-109818475*	*AX-109929319*	3.3566	9.5863	0.8289
	*QFSPN.sicau-7D.1*	BLUP	7D	106	*AX-110502471*	*AX-111932357*	2.781	4.1159	-0.366

PVE, phenotypic variation explained; LOD, logarithm of odds; Add additive effect (positive values indicate that alleles from 10-A are increasing the trait scores, and negative values indicate that alleles from B39 are increasing the trait scores); BLUP best linear unbiased prediction.

Eighteen QTLs for FSPN were co-localized on chromosomes 1B (one QTL), 2A (three), 2D (three), 3B (one), 3D (two), 4B (two), 5A (three), 6D (one), 7B (one), and 7D (one). *QFSPN.sicau-2D.1* was detected in 10 environments (E1, E2, E3, E4, E5, E6, E7, E8, E9, and BLUP) with LOD values of 3.23–11.68. This QTL was located between *AX-109465277* and *AX-109785183*, explaining 6.80%–19.83% of the phenotypic variance, and the phenotypic contribution rate was >10% in eight environments. The additive effect of the QTL was derived from 10-A. *QFSPN.sicau-2D.3* was detected in six environments (E2, E3, E5, E6, E7, and BLUP) with LOD values of 2.99–8.94, explained 7.77%–15.92% of the phenotypic variation, and its additive effects were derived from 10-A. *QFSPN.sicau-2A.3* was detected in four environments (E2, E8, E9, and BLUP) and explained 4.08%–8.94% of the phenotypic variation. The remaining QTLs were detected in a single environment and explained 3.12%–18.27% of the phenotypic variation. Among these QTLs, *QFSPN.sicau-3B.1*, *QFSPN.sicau-3D.2*, *QFSPN.sicau-4B.2*, and *QFSPN.sicau-7D.1* alleles were derived from B39. The remaining QTL alleles were derived from 10-A.

### 3.5 Repositioning with new markers

**Figure 2:**
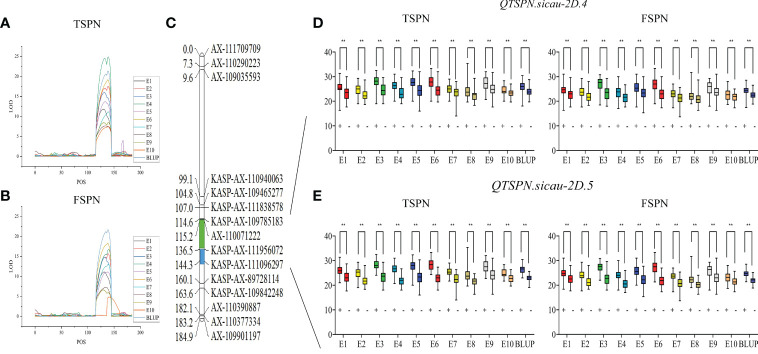
Genetic map of the major quantitative trait locus (QTL) *QTSPN.sicau-2D.4* and *QTSPN.sicau-2D.5* and its effect. LOD value of TSPN QTL **(A)** and LOD value of FSPN QTL **(B)** on chromosome 2D among ten environmental and BLUP values. Genetic map integrated with the developed Kompetitive Allele-Specific PCR (KASP) marker **(C)**. The green and blue areas represent the major QTL *QTSPN.sicau-2D.4* and *QTSPN.sicau-2D.5*, respectively. The total spikelet number per spike (TSPN) and fertile spikelet number per spike (FSPN) were shown as box plots calculated after grouping the 10-A/B39 population into two classes based on the KASP marker of the QTL *QTSPN.sicau-2D.4* **(D)** and *QTSPN.sicau-2D.5* **(E)**; + and - indicate the lines with and without positive alleles of QTL; ** represents significance at P < 0.01.

To narrow the QTL mapping range, based on the results of the wheat 55K SNP array, eight pairs of KASP markers were developed on either side and within the interval for *QTSPN.sicau-2D.1* (the marker information is shown in **Supplementary Table 5**). Combining the genotyping results for the eight pairs of KASP markers and the wheat 55K array genotype identification results for the 10-A/B39 population, QTL mapping was repeated for TSPN and FSPN (**Table 3**; **Figure 2**), which were mapped to two new QTLs for TSPN (*QTSPN.sicau-2D.4* and *QTSPN. sicau-2D.5*) and two new QTLs for FSPN (*QFSPN.sicau-2D.4* and *QFSPN.sicau-2D.5*). Detected in five environments (E1, E3, E4, E5, and E8), *QTSPN.sicau-2D.4* explained 21.76%–38.49% of the phenotypic variation with LOD values of 7.20–24.49 and was located between *AX-110071222* and *KASP-AX-111956072*. *QTSPN.sicau-2D.5* was detected in seven environments (E2, E4, E6, E7, E9, E10, and BLUP), explaining 20.59%–44.83% of the phenotypic variation with LOD values of 7.47–24.88, and was located between *KASP-AX-111956072* and *KASP-AX-111096297*. *QFSPN.sicau-2D.4* was detected in four environments (E1, E3, E8, and E9) and explained 17.19%–34.81% of phenotypic variation with LOD values of 6.62–14.80. *QFSPN.sicau-2D.5* was detected in seven environments (E2, E4, E5, E6, E7, E10, and BLUP) and explained 13.97%–45.90% of the phenotypic variation with LOD values of 4.83–21.66. The additive effects of these QTLs were all derived from 10-A.

**Table 3:** QTL mapping for the total spikelets per spike and the fertile spikelets per spike in wheat from “10-A×B39”RIL population at 55K SNP chip and KASP.

Trait	QTLs	Environment	Chromosome	Position (cM)	LeftMarker	RightMarker	LOD	PVE(%)	Add
TSPN	*QTSPN.sicau-2D.4*	E1	2D	130	*AX-110071222*	*KASP-AX-111956072*	11.8104	27.9482	1.7326
		E3	2D	131			15.9684	37.3348	2.2036
		E4	2D	132			24.4922	27.4535	2.581
		E5	2D	133			13.6635	38.4968	1.9953
		E8	2D	133			7.6088	21.7635	1.2856
	*QTSPN.sicau-2D.5*	E2	2D	139	*KASP-AX-111956072*	*KASP-AX-111096297*	17.4804	39.6025	1.7606
		E4	2D	139			24.8812	24.7605	2.4819
		E6	2D	138			19.1765	43.6354	2.2869
		E7	2D	139			11.1232	27.7103	1.3785
		E9	2D	139			8.4775	21.6894	1.6699
		E10	2D	139			7.4745	20.5918	0.9673
		BLUP	2D	138			21.307	44.8377	1.6596
FSPN	*QFSPN.sicau-2D.4*	E1	2D	131	*AX-110071222*	*KASP-AX-111956072*	11.091	27.1352	1.6753
		E3	2D	133			14.8006	34.8128	2.1666
		E8	2D	135			7.2421	20.9322	1.1784
		E9	2D	129			6.6185	17.1941	1.7141
	*QFSPN.sicau-2D.5*	E2	2D	139	*KASP-AX-111956072*	*KASP-AX-111096297*	15.6585	36.6052	1.6686
		E4	2D	139			16.7048	40.2418	1.9231
		E5	2D	138			11.0885	29.4836	1.7408
		E6	2D	138			18.3061	42.5122	2.4336
		E7	2D	139			12.991	31.778	1.4912
		E10	2D	141			4.8334	13.9702	0.8328
		BLUP	2D	138			21.6638	45.8991	1.4604

PVE, phenotypic variation explained; LOD, logarithm of odds; Add additive effect (positive values indicate that alleles from 10-A are increasing the trait scores, and negative values indicate that alleles from B39 are increasing the trait scores); BLUP best linear unbiased prediction.

### 3.6 Validation of *QTSPN.sicau-2D.4* and *QTSPN.sicau-2D.5* in different genetic backgrounds

**Figure 3:**
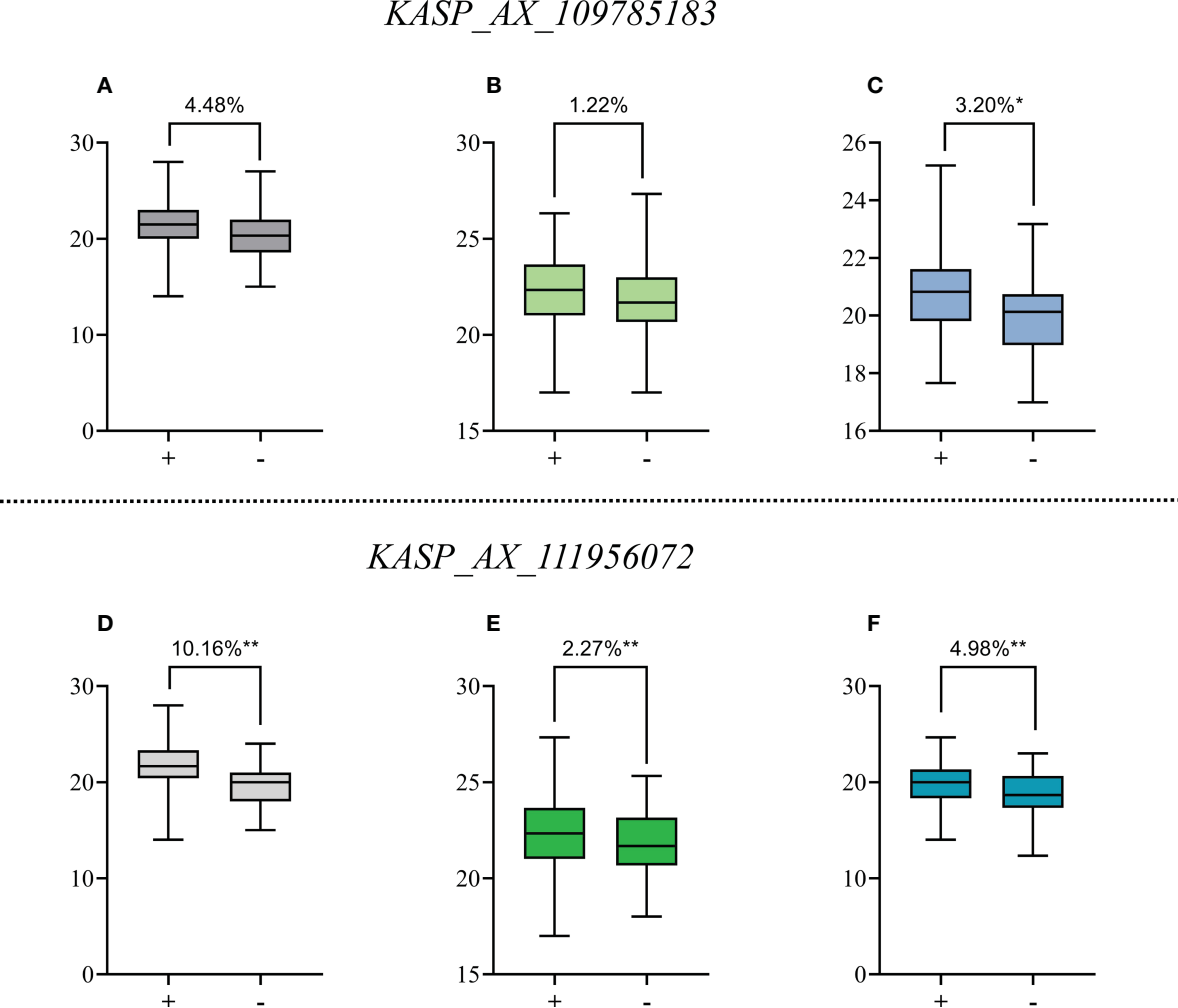
Effects of *QTSPN.sicau-2D.4* and *QTSPN.sicau-2D.5* on total spikelet number per spike (TSPN) in the 10-A/BE89 population **(A, D)**, 10-A/CN16 population **(B, E)** and CD population **(C, F)**. + and - indicate the lines with and without positive alleles of *QTSPN.sicau-2D.4* and *QTSPN.sicau-2D.5*; * and ** represent significance at P < 0.05 and P < 0.01, respectively.

The *KASP_AX_109785183* marker closely linked to QTSPN.sicau-2D.4, and the *KASP_AX_111956072* marker closely linked to *QTSPN.sicau-2D.5* both detected polymorphism between 10-A and B39. Thus, the 10-A/BE89 population, 10-A/CN16 population, and the CD population of Sichuan wheat accessions were employed to verify *QTSPN.sicau-2D.4* and *QTSPN.sicau-2D.5* in different genetic backgrounds (**Figure 3**).

The marker *KASP_AX_109785183* successfully genotyped all populations. Among the CD population, 83 lines were type A from 10-A, 71 were type B from B39, and 27 were heterozygous. Significant differences in TSPN between the parental types were detected (P < 0.05). TSPN of the A-type was increased by 3.20% compared with the B-type. In the 10-A/BE89 population, 68 lines were A-type-, 56 were type-B, and ten were heterozygous. The TSPN of the parent types was not significantly different (P>0.05). In the 10-A/CN16 population, 88 lines were A-type, 75 were B-type, and 29 were heterozygous. The TSPN of the parent types was not significantly different (P > 0.05), and the TSPN of the A-type was increased by 1.22% compared with the B-type.

The *KASP_AX_109785183* marker successfully genotyped all three populations. Among the CD population, 42 lines were A-type, 172 were B-type, and 15were heterozygous. The difference in TSPN between the parental types was strongly significant (P < 0.01). The TSPN of the A-type was increased by 4.98% compared with the B-type. In the 10-A/BE89 population, 68 lines were A-type, 58 were B-type, and 15 were heterozygous. The difference in TSPN between the parental types was strongly significant (P < 0.01), and the TSPN of the A-type was increased by 10.16% compared with the B-type. In the10-A/CN16 population, 91 lines were A-type, 77 were B-type, and 24 were heterozygous. The difference in TSPN between the parental types was highly significant (P < 0.01), and the TSPN of the A-type was increased by 2.27% compared with the B type.

### 3.7 Narrowing QTL intervals by exon capture sequencing

**Figure 4:**
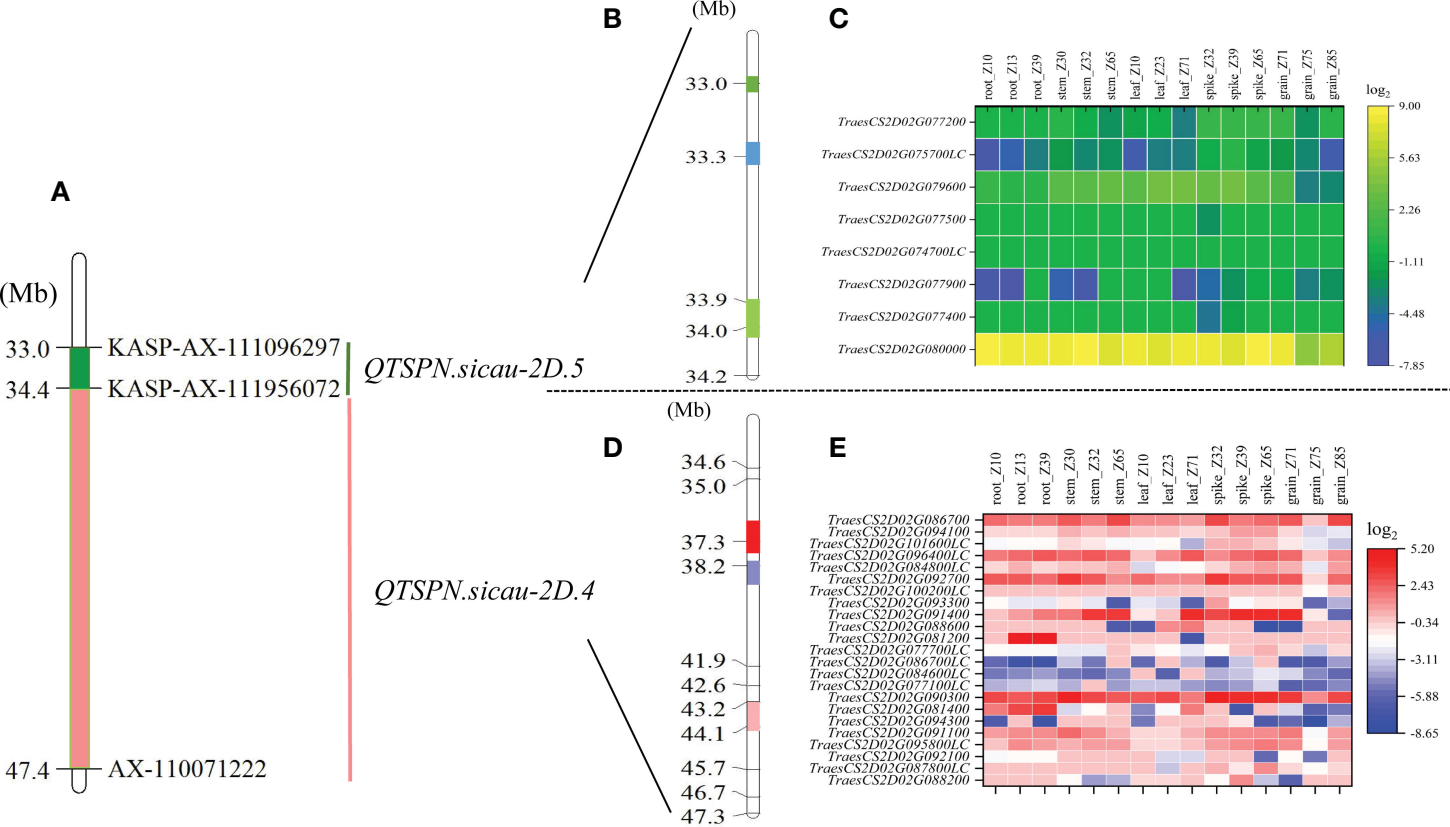
Physical maps and exon capture results of major quantitative trait loci (QTL) *QTSPN.sicau-2D.4* and *QTSPN.sicau-2D.5*. Physical map integrated with the developed Kompetitive Allele-Specific PCR (KASP) marker **(A)**. The green area is the interval of the major QTL *QTSPN.sicau-2D.5* **(B)**. The three colored areas represent the three hot spots, and the numbers on the left are the physical locations. **(C)** is the expression map of the predicted genes within *QTSPN.sicau-2D.5* in different wheat tissues at different stages. The Pink area is the interval of the major QTL *QTSPN.sicau-2D.4* **(D)**. The three colored areas represent the three hot spots, and the numbers on the left are the physical locations. The expression map of the predicted genes within *QTSPN.sicau-2D.4* in different wheat tissues at different stages **(E)**.

A total of 203 SNP sites were captured in the interval of *QTSPN.sicau-2D.4/QFSPN.sicau-2D.4* by bulk-segregant analysis–exon capture sequencing (**Figure 4**). The results revealed that 168 mutated SNPs were concentrated in three intervals. The SNPs were mapped to the IWGSC RefSeq v1.1 genome assembly. Thirty-two mutation sites were located between 37.25 and 37.52 Mb, accounting for 15.76% of the total mutation sites. Eighty-three mutation sites were located between 38.20 and 38.79 Mb, accounting for 40.88% of the total mutation sites. Fifty-three mutation sites were located between 43.22 and 43.90 Mb, accounting for 26.11% of the total number. KASP markers were developed based on the SNP mutation hot spots in the candidate region, namely *KASP_35677660* and *KASP_45881230* (**Supplementary Table 5**), and genotyping of the 10-A/B39 population was performed. Genotyping was successful for both markers. Using *KASP_35677660*, 85 lines with the 10-A allele and 48 with the B39 allele were identified. Three lines were heterozygous and genotyping failed for the remaining 16 lines. The difference in TSPN between the parental types was strongly significant (P < 0.01). Using *KASP_45881230*, 81 lines with the 10-A allele and 56 with the B39 allele were identified. Two lines were heterozygous, and genotyping failed for the remaining 13 lines. The difference in TSPN between the parental types was significant (P < 0.05). *KASP_35677660* and *KASP_45881230* were tightly linked markers for *QTSPN.sicau-2D.4*.

Eighteen mutated SNP sites were captured in the interval for *QTSPN.sicau-2D.5* and *QFSPN.sicau-2D.5* (**Figure 4**). Seventeen mutation sites were concentrated in three intervals, with four mutation sites located between 32.97 and 33.04 Mb, accounting for 22.22% of the total mutation sites. Eight mutation sites were located between 33.31 and 33.32 Mb, accounting for 44.44%. Five mutation sites were located between 33.89 and 34.02 Mb, accounting for 27.78% of the total.

### 3.8 Relationship between QTSPN.sicau-2D.4 and QTSPN.sicau-2D.5

**Figure 5:**
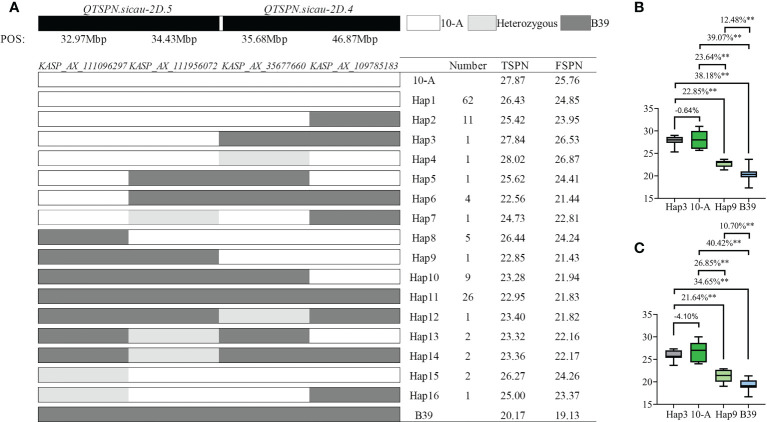
Genotyping map of *QTSPN.sicau-2D.4* and *QTSPN.sicau-2D.5* loci in 10-A/B39 population. **(A)** is different types of two QTLs in the 10-A/B39 population; the total spikelet number per spike **(B)** and fertile spikelet number per spike **(C)** in tye3, tye 9, 10-A, B39 were compared. ** represents significance at P < 0.01.

The combination of four tightly linked KASP markers, i.e., *KASP_AX_111096297* and *KASP_AX_111956072* for *QTSPN.sicau-2D.5*, and *KASP_35677660* and *KASP_AX_109785183* for *QTSPN.sicau-2D.4*, was applied to genotype the 10-A/B39 population (**Figure 5**). A total of 16 combinations were detected, indicating that there was no co-segregation between *QTSPN.sicau-2D.4* and *QTSPN.sicau-2D.5*.

In the genotyping results with the four pairs of KASP markers for the 10-A/B39 population, the haplotype represents the combination of the allelic variation of the four markers in 10-A and B39. The phenotype data yielded the following results: haplotype 1 of 10-A comprised 62 lines, and haplotype 11 of B39 comprised 26 lines. The spikelet number of haplotype 1 was strongly significantly higher than that of haplotype 11 (P < 0.01). The number of spikelets of haplotype 3 lines exceeded that of 10-A in 10 environments, and the TSPN of haplotype 9 lines was significantly higher than that of B39 in ten environments (P < 0.01). The TSPN of haplotype 3 was significantly higher than that of haplotype 9 in the 10 environments. About TSPN, the ranking haplotype 3 > 10-A > haplotype 9 > B39 was observed, indicating that the combination of *QTSPN.sicau-2D.5* with the 10-A allele and *QTSPN.sicau-2D.4* with the B39 allele resulted in the highest number of spikelets.

### 3.9 Potential candidate genes for *QTSPN.sicau-2D.4* and *QTSPN.sicau-2D.5*

The mutated SNP sites co-captured in the *QTSPN.sicau-2D.4*/*QFSPN.sicau-2D.4* interval were individually aligned to the wheat genome. In total, 148 mutation sites corresponded to 23 candidate genes in the wheat genome. Twenty-three genes were functionally annotated (**Supplementary Table 6**), mainly proteins and ubiquitin. A GO enrichment analysis was performed on the 23 genes. Six genes (*TraesCS2D02G081400*, *TraesCS2D02G086700*, *TraesCS2D02G088200*, *TraesCS2D02G090300*, *TraesCS2D02G092100*, and *TraesCS2D02G094100*) were involved in response to biological stimuli, anchoring components of membranes, cytoskeleton, ubiquitin-like modifier activating enzyme activity, toxin activity, negative regulation of translation, ribosomal RNA N-glycosylase activity, serine-type peptidase activity, and calmodulin binding. The 18 mutated SNP sites captured in the *QTSPN.sicau-2D.5*/*QFSPN.sicau-2D.5* interval were aligned to the wheat genome, and 13 mutation sites were detected, corresponding to the exons of eight candidate genes. Functional annotation of the eight genes in wheat genome assembly version 1.1 indicated comprised proteins, reductases, and subunits. The GO functional annotation analysis of the eight genes (**Table 4**) revealed two genes, *TraesCS2D02G079600 (Ppd-D1)* and *TraesCS2D02G080000*, were annotated with four functions, namely negative regulation of long-day photoperiod phenomenon, phosphorescence relay signal transduction system, peroxidase activity, and response to oxidative stress.

**Table 4:** GO annotation results of 31 target genes.

GO term	Description	P-value	FDR	Number	Group	GeneID
GO:0006952	defense response	0.00207	0.0139	2	biological_process	*TraesCS2D02G081400/TraesCS2D02G090300*
GO:0030036	actin cytoskeleton organization	0.00659	0.0139	1	biological_process	*TraesCS2D02G088200*
GO:0010215	cellulose microfibril organization	0.00913	0.0139	1	biological_process	*TraesCS2D02G092100*
GO:0016049	cell growth	0.00935	0.0139	1	biological_process	*TraesCS2D02G092100*
GO:0006464	cellular protein modification process	0.0108	0.0139	1	biological_process	*TraesCS2D02G086700*
GO:0009607	response to biotic stimulus	0.0119	0.0139	1	biological_process	*TraesCS2D02G081400*
GO:0031225	anchored component of membrane	0.00745	0.0149	1	cellular_component	*TraesCS2D02G092100*
GO:0005856	cytoskeleton	0.0183	0.0183	1	cellular_component	*TraesCS2D02G088200*
GO:0008641	ubiquitin-like modifier activating enzyme activity	0.00606	0.0223	1	molecular_function	*TraesCS2D02G086700*
GO:0090729	toxin activity	0.00744	0.0223	1	molecular_function	*TraesCS2D02G090300*
GO:0017148	negative regulation of translation	0.0238	0.0238	1	biological_process	*TraesCS2D02G090300*
GO:0030598	rRNA N-glycosylase activity	0.0125	0.0249	1	molecular_function	*TraesCS2D02G090300*
GO:0008236	serine-type peptidase activity	0.0177	0.0251	1	molecular_function	*TraesCS2D02G094100*
GO:0005516	calmodulin binding	0.0209	0.0251	1	molecular_function	*TraesCS2D02G081400*
GO:0048579	negative regulation of long-day photoperiodism, flowering	0.000948	0.00284	1	biological_process	*TraesCS2D02G079600*
GO:0000160	phosphorelay signal transduction system	0.0129	0.0194	1	biological_process	*TraesCS2D02G079600*
GO:0004601	peroxidase activity	0.0238	0.0238	1	molecular_function	*TraesCS2D02G080000*
GO:0006979	response to oxidative stress	0.038	0.038	1	biological_process	*TraesCS2D02G080000*

The expression levels of 31 candidate genes were predicted in the roots, stems, leaves, spikes, and seeds of wheat during three spike developmental stages using WheatOmics 1.0 (**Figure 4**). The genes *TraesCS2D02G090300*, *TraesCS2D02G091400*, *TraesCS2D02G080000*, and *TraesCS2D02G079600* had higher predicted expression levels in the spike in the three developmental stages. Homologous gene alignment for the target genes with the TriticeaeGeneTribe database detected homologs in rice and *Arabidopsis* (**Table 4**). Most of the genes corresponded to multiple homologous genes in rice and *Arabidopsis*, and the consistency was 22.16%–90.00%. Moreover, a significant difference in spikelet number between the parental types was observed (P <0.01) using the molecular markers of the *TraesCS2D02G079600 (Ppd-D1)* gene.

### 3.10 Cloning and analysis of *Ppd-D1*

**Figure 6:**
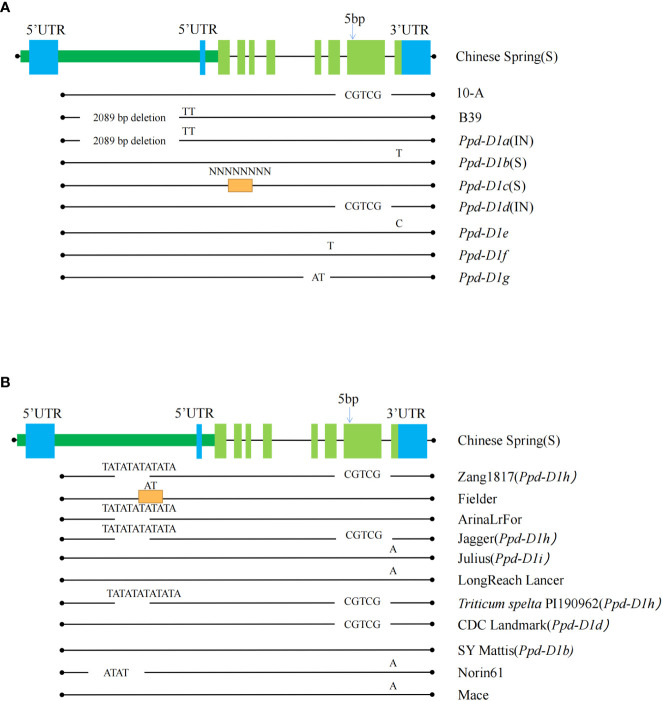
The sequence characteristics of the pseudo-response regulator (*Ppd-D1*) gene. Comparing the *Ppd-D1* genes among 10-A, B39, and seven other allelic variation types **(A)** and the other types of variation of the *Ppd-D1* gene in different wheat varieties **(B)**. Allelic variant type sequences found from other cultivars are shown in the black line below each ‘Chinese Spring’ sequence. Black dots indicate the edges. Chinese spring sequence is shown as rectangles (dark green long rectangle indicates promoter region, blue rectangle for 5’ UTR and 3’ UTR regions. Light green rectangles indicate the exon regions). Regions with missing lines show deletions of the sequence, and orange rectangles indicate the presence of an insertion in that region. Specific deletion and inserted sequences are annotated above them. S indicates that this allelic variant type is photoperiod-sensitive, and IN is photoperiodic-insensitive. The SNP shows the position of the single-nucleotide variants.

The 5981 bp sequence, including the coding region of *Ppd-D1* was amplified using all coding regions of the NCBI website (**Figure 6**). Fragments of 5976 bp (10-A) and 3892 bp (B39) were amplified. Alignment of the 10-A and B39 amplified sequences with the sequence from Chinese Spring revealed that the *Ppd-D1* gene shares eight exon regions. The sequence from B39 lacked a 2089 bp fragment in the promoter region, and the sequence from 10-A contained a five bp deletion in exon 7. These results indicated that the *Ppd-D1* gene from B39 and 10-A was the *Ppd-D1a* allele and *Ppd-D1d* allele, respectively. Translation of the sequence of B39 is affected by the large deletion in the promoter region, which significantly affects the expression of *Ppd-D1a*; the expressed protein of 10-A is altered owing to the five bp deletion in exon seven, and thus it may not perform its original function.

After BLAST alignment of *Ppd* gene sequences in NCBI, seven mutation types in the Ppd-D1 gene of hexaploid wheat were detected. Chinese Spring had the *Ppd-D1b* (accession: KJ147478) type, which is light-sensitive. Compared with the complete sequence of *Ppd-D1*, *Ppd-D1a* (accession: KJ147477) contained the 2089 bp deletion in the promoter region, *Ppd-D1c* (accession: KJ147483) included insertion of an unknown sequence in exon 2, *Ppd-D1d* (accession: KJ147481) had a five bp deletion in exon 7, *Ppd-D1e* (accession: KJ147482) has a single-base mutation in exon 8, *Ppd-D1f* (accession: KJ147479) included an AT base mutation between exons 5 and 6, and *Ppd-D1h* (accession: KJ147484) contained an AT deletion in exon 5.

To further understand the variation of *Ppd-D1*, the WheatOmics platform (http://202.194.139.32/) was used to compare the *Ppd-D1* gene in different wheat varieties. In addition to the seven mentioned above types, five variant types were detected, including an AT deletion in the promoter. This mutation directly affects the function of a protein type showing reduced photoperiod sensitivity, similar to the *Ppd-D1a* mutant type, and thus could be used as an alternative allele to *Ppd-D1a*. The five bp deletion in exon 7 of *Ppd-D1* was observed in various wheat cultivars, including Zang1817, Jagger, *Triticum spelta* PI190962, and CDC Landmark. Among these cultivars, CDC Landmark is of the *Ppd-D1d* type. Thus, the variation in *Ppd-D1* tended to be photoperiod-insensitive in the wheat materials examined.

## 4 Discussion

### 4.1 Two major QTLs for TSPN and FSPN

To date, QTLs controlling spikelet number have been detected on all 21 chromosomes of wheat; for example, the QTLs *Xwmc181.1-Xaf12d*, *Xaf12–Xcfd239*, *Xcfd267–Xmag3596*, *Xbarc228–Xwmc181.1*, and *QTsn.czm-2D.3* are located on chromosome 2D (Ma et al., 2007; Cui et al., 2012; Zhou et al., 2017; Li et al., 2021). Those QTLs on the long arm of chromosome 2D are mainly concentrated between 480 and 650 Mb (Li et al., 2002; Zhou et al., 2017; Deng et al., 2019; Li et al., 2021; Saini et al., 2022). The reported QTLs on the short arm of 2D include *Xbcd611–Xgwm484*. The present positioning results indicated that most QTLs were concentrated between 10.00 and 43.00 Mb. The QTLs identified in this study located on the short arm of 2D were *QTSPN.sicau-2D.4* (chr2D: 34.43–47.43 Mb) and *QTSPN.sicau-2D.5* (chr2D: 32.97–34.43 Mb).

Deng et al. (2019) detected *QTSS.sicau-2D.2* in four environments and BLUP values, and the QTL explained 12.8%–18.5% of the phenotypic variation. We mapped the resulting QTL *QTSPN.sicau-2D.4*, similar to *QTSS.sicau-2D.2*, and observed that *QTSPN.sicau-2D.4* explained 21.76%–38.49% of the phenotypic variation in five environments. Ma et al. (2019) constructed a RIL population from 20828 and CN16, and detected *QSns.sau-2D* in six environments and BLUP values, explaining 10.16%–45.68% of the phenotypic variation. In addition, these authors detected Q*TSPN.sicau-2D.5* in seven environments and BLUP values, similar to *QSns.sau-2D*, which explained 20.59%–44.83% of the phenotypic variation. In previous studies, only one similar QTL on chromosome 2D was mapped in each population. In the present study, we detected two similar QTLs in one RIL population, suggesting that two QTLs in this region control spikelet number, providing an opportunity to investigate the regulation and interaction of these two QTLs. These results contribute to elucidating the regulatory mechanism of spikelet number in wheat.

### 4.2 Pyramiding of *QTSPN.sicau-2D.4* and *QTSPN.sicau-2D.5* for trait improvement

Allele aggregation is an effective method to enhance phenotypic traits in wheat breeding (Tu et al., 2021). Previous studies compared the relationship between QTLs mapped for different traits (Li et al., 2021), and some compared the relationship between QTLs on different chromosomes obtained by mapping the same trait (Shao et al., 2018). In the present study, we investigated the relationship between *QTSPN.sicau-2D.4* and *QTSPN.sicau-2D.5* and observed that the two QTLs had simultaneous effects in only one environment among the ten environments. We speculated that *QTSPN.sicau-2D.4* and *QTSPN.sicau-2D.5* can only be co-expressed in specific environments. In a previous study (Deng et al., 2019), the TSPN of the parents SHW-L1 and Chuanmai 32 was 19.6–21.6 and 19.6–25. In some environments, the differences in TSPN between the parents were small or non-significant. The reason may be that the effect of *QTSS.sicau-2D.2* is only detected in a specific environment, and the additive effect is small. The present study showed that a tightly linked marker for *QTSPN.sicau-2D.4* could be used successfully to genotype the 10-A/BE89 and the 10-A/CN16 population, but not significant. This result may be because the environment in which these two populations were grown did not meet the conditions necessary to express *QTSPN.sicau-2D.4*. The present results showed the effect on wheat spikelet number of genes located in *QTSPN.sicau-2D.4* was a lower than that of genes located in *QTSPN.sicau-2D.5*.

Genotyping with the four closely linked KASP marker pairs of the 10-A/B39 population revealed 16 haplotypes of progeny populations, indicating no co-segregation between the two QTLs. About TSPN, the types were ranked as haplotype 3 > 10-A > haplotype 9 > B39. Thus, we speculated that *QTSPN.sicau-2D.4* and *QTSPN.sicau-2D.5* both include genes that increase TSPN, but the expression of genes in *QTSPN.sicau-2D.4* may affect the expression of genes in *QTSPN.sicau-2D.5*, and thus the haplotype 3 spikelet number was higher than that of the 10-A type. However, owing to the small number of RILs used, further study of the relationship between the two QTLs will require an increase in the number of RILs or the construction of near-isogenic lines.

### 4.3 Exon capture shortens the target region

Previous studies have used exon capture to identify wheat mutants resistant to yellow rust and leaf rust and to identify an SNP mutation in the *Lr21* gene (Harrington et al., 2019). Exome capture has also been used to study the processes of gene introgression and gene improvement in wheat (He et al., 2019). In the current study, to narrow the target QTL interval, bulk-segregant analysis–exon capture sequencing was used to detect six hot spots in *QTSPN.sicau-2D.4* and *QTSPN.sicau-2D.5*. The mutation sites in these six regions were much higher than those in other regions. Therefore, we believed that the probability that the target gene is in these six regions was higher than its location in other regions, so the subsequent experiments considered these six regions as the main target interval to narrow the scope of the target interval. By comparing the physical locations of the detected mutated SNPs, we identified 31 candidate genes. The SNP loci of these genes differed between the parents and offspring, so that these genes will be the focus of a future study. Li et al. (2021) identified target genes by comparing their expression levels in different tissues. Therefore, we analyzed the expression levels of the 31 candidate genes in different tissues at three stages of spike development. *TraesCS2D02G080000* and *TraesCS2D02G090300* were highly expressed in the spike. *TraesCS2D02G092700* was highly expressed in the early and weakly expressed in the final stages of spike development. These results will also help the further development of the future study.

### 4.4 Candidate genes of QTSPN.sicau-2D.4/QFSPN.sicau-2D.4

Through exon capture and sequencing, 203 mutant SNP sites were captured in the *QTSPN.sicau-2D.4*/*QFSPN.sicau-2D.4* interval. Of the 203 mutation sites, 148 corresponded to exon sequences of 23 genes, and the remaining mutation sites were located in intronic regions. Compared with the five target genes obtained in previous studies (Deng et al., 2019), one target gene, *COBL7*, was typical. *COBL7* is a homologous gene of *TraesCS2D02G092100* and affects pistil development and function (Scutt et al., 2003; Izadi et al., 2021). The homologous genes of *TraesCS2D02G077100LC* are *OsMYB3R-2* and *ATU2AF65A*. Overexpression of *OsMYB3R-2* increases tolerance to low temperature, drought, and salt stress in Arabidopsis (Dai et al., 2007). *ATU2AF65A* is a splicing factor involved in regulating of *ABA*-mediated flowering time in *Arabidopsis* (Park et al., 2017; Cavallari et al., 2018). The homologous gene of *TraesCS2D02G081200* is the *OsYSL* family gene, which is mainly responsible for iron uptake during the early growth of seedlings (Kobayashi et al., 2009). *TraesCS2D02G086700* is ubiquitin activating enzyme 2, and its homolog is *SAE2*, which is involved in plant meiosis (Uanschou et al., 2007). The homologous gene of *TraesCS2D02G095800LC* is *ACD11*, a mediator of phytoceramide levels (Simanshu et al., 2014). The *TraesCS2D02G093300* homolog is *OsRFP*, a positive regulatory gene that responds to salt stress by regulating Na+ uptake (Kim et al., 2021). *TraesCS2D02G101600LC* is a leucine-rich repeat receptor-like kinase involved in specifying of anther cell identity, controlling of early sporogenesis and development, and anther wall formation (Nonomura et al., 2003). These genes may be the candidate genes that control spikelet number and require further screening.

### 4.5 Candidate genes for QTSPN.sicau-2D.5/QFSPN.sicau-2D.5

In the *QTSPN.sicau-2D.5*/*QFSPN.sicau-2D.5* interval, 18 SNP sites were captured and aligned to the wheat genome. Thirteen of the 18 SNP sites corresponded to exons of eight genes. The homologous genes of *TraesCS2D02G077200* are *TRM19*, *TRM20*, and *TRM21* in *Arabidopsis*. Modified nucleosides in the *TRM* gene in *Arabidopsis* are essential for protein translation. For example, *TRM5* affects the growth of *Arabidopsis*, is associated with delayed flowering, and affects the abundance of photosynthetic proteins (Guo et al., 2019). *TraesCS2D02G077400* and *TraesCS2D02G077500* both contain the *DUF569* domain of the unknown function. In *Arabidopsis*, *AtDUF569* negatively regulates biotic stress responses and differentially regulates plant growth under nitrogen oxidative stress (Nabi et al., 2020). *TraesCS2D02G077900* contains the structure of the heat shock protein DnaJ, identified initially as a heat shock protein in *E*. *coli*. It belongs to a highly diverse family of molecular chaperones. It plays an important role in the regulating protein folding, various physiological activities, and plant development and stress response (Cyr and Langer, 1994; Li et al., 2020; Zhang et al., 2021; Jin et al., 2022). *TraesCS2D02G079600* is a *Ppd-D1* gene that affects plant growth and development and is an important photosensitive gene. The *Ppd-D1*-derived InDel marker showed that the *QSns.sau-2D* of total spikelet number per spike was not associated with *Ppd-D1 (* Ma et al., 2019). *TraesCS2D02G080000* is an ascorbate peroxidase involved in the homeostasis of reactive oxygen species, chloroplast protection, carbohydrate metabolism, plant structure, fertility maintenance, and other physiological activities. Its homologous gene in rice is *APX2*, which is involved in hydrogen peroxide homeostasis, chloroplast protection, plant configuration, and fertility maintenance (Wu et al., 2018). These eight genes will be the focus of future research.

### 4.6 Effects of *Ppd-D1* on the growth period of wheat

The *QTSPN.sicau-2D.5* interval partially overlaps with the physical interval of *Ppd-D1* (photoperiod). *Ppd-D1* is a photoperiod-sensitive gene closely related to wheat adaptability to the environment and affects plant flowering and higher traits. However, the genetic mechanism of how *Ppd-D1* affects the spikelet number in wheat has not been reported. Multiple allelic variant types of the *Ppd-D1* gene are currently known (Guo. et al., 2010). Those relatively familiar in Asian wheat germplasm are *Ppd-D1a*, *Ppd-D1b*, and *Ppd-D1d*. Interaction of *Ppd-D1* and vernalization genes (*Vrn1*, *Vrn2*, and *Vrn3*) affects the wheat reproductive period (Foulkes et al., 2004; Mokanu and Fayt, 2008; Quraishi et al., 2011). The interaction of *Ppd-D1* with dwarfing genes (*Rht8*, *Rht-B1b*, and *Rht-D1b*) affects plant height (Flintham et al., 1997; Reynolds et al., 2012; Cane et al., 2013). Beales et al. (2007) isolated the wheat *Ppd-A1*, *Ppd-B1*, and *Ppd-D1* genes using a wheat bacterial artificial chromosome library. Sequence analysis revealed that *Ppd-D1a* contained a 2089 bp deletion upstream of the coding region. This deletion in *Ppd-D1b* is crucial to the origin of the photoperiod-insensitive allele *Ppd-D1a*. The deletion not only changes the gene’s expression pattern but also changes the expression of the downstream genes *CO1* and *FT*. The *Ppd-D1* gene plays an important role in forming paired spikelets by regulating flower-promoting signals (*FT*) strength during early reproductive development (Boden et al., 2015). The 5-bp-deletion mutation of *Ppd-D1d* in exon seven leads to a frameshift of the amino acid reading frame, producing a functional protein but destroying the CCT domain (CCT plays a critical role in the flowering regulation of sunlight length). CCT plays an important role in the day-length regulation of flowering, and the CCT domain is required to respond to light. The allele *Ppd-D1d* is equivalent to an alternative allele of *Ppd-D1a*, and the role of *Ppd-D1d* is to delay heading relative to *Ppd-D1a* (Nishida et al., 2013).

In this study, the deletion of a large fragment in the promoter region of B39 (*Ppd-D1a*) meant that the *Ppd-D1a* mutant was phenotypically insensitive to photoperiod and early flowering regardless of day length. In contrast, the 10-A (*Ppd-D1d*) accession attains heading later and has a greater number of spikelets, and a statistically significant difference in TSPN between the two variant types was observed. Previous research indicated that the *Ppd-D1a* and *Ppd-D1d* mutants have little effect on wheat yield (Eagles et al., 2014) and that *Ppd-D1d*, as an alternative allele of the *Ppd-D1a* variant, should have less of a statistical impact on phenotypes. Thus, it is speculated that additional genes are in the positioning interval and that their interaction with the *Ppd-D1* gene affects the number of spikelets. Further exploration of whether other genes in the QTL interval affect the number of spikelets in wheat is required.

## Data availability statement

The original contributions presented in the study are included in the article/**Supplementary Material**. Further inquiries can be directed to the corresponding author.

## Author contributions

XY: Visualization, Writing-original draft. YY: Visualization, Writing-original draft. JinW: Visualization, Writing-original draft. ZL: Investigation, Data curation. JL: Investigation, Data curation. YC: Investigation, Data curation. GC: Methodology. JM: Methodology. ZP: Methodology. YP: Formal analysis, Software. PQ: Methodology. YL: Formal analysis, Software, Resources. QJ: Formal analysis, Software, Resources. JirW: Formal analysis, Software, Resources. YW: Conceptualization. YZ: Writing-review and editing. WL: Conceptualization, Funding acquisition, Project administration, Writing-review and editing, Supervision. All authors contributed to the article and approved the submitted version.

## Supplementary material

Supplementary Figure 1 Phenotypic performances, distribution, and correlation coefficients for total spikelet number per spike (TSPN) **(A)** and fertile spikelet number per spike(FSPN) **(B)** of parents and 10-A/B39 lines in ten environments and their corresponding best linear unbiased prediction (BLUP) values; *** represents significance at P < 0.001.

Supplementary Figure 2 Schematic representation of the syntenic relationships between a given marker in wheat genetic and physical maps. 1A to 7D represent the 21 wheat chromosomal genetic maps released in this paper; Phy-1A to Phy-7D represent the 21 wheat chromosomal physical maps.
