# Supplementary figures and images for: Identification and validation of two major QTLs for spikelet number per spike in wheat (Triticum aestivum L.)
Source: Front Plant Sci. 2023 May 10;14:1144486. doi: 10.3389/fpls.2023.1144486 (PMC10208070; doi:10.3389/fpls.2023.1144486)

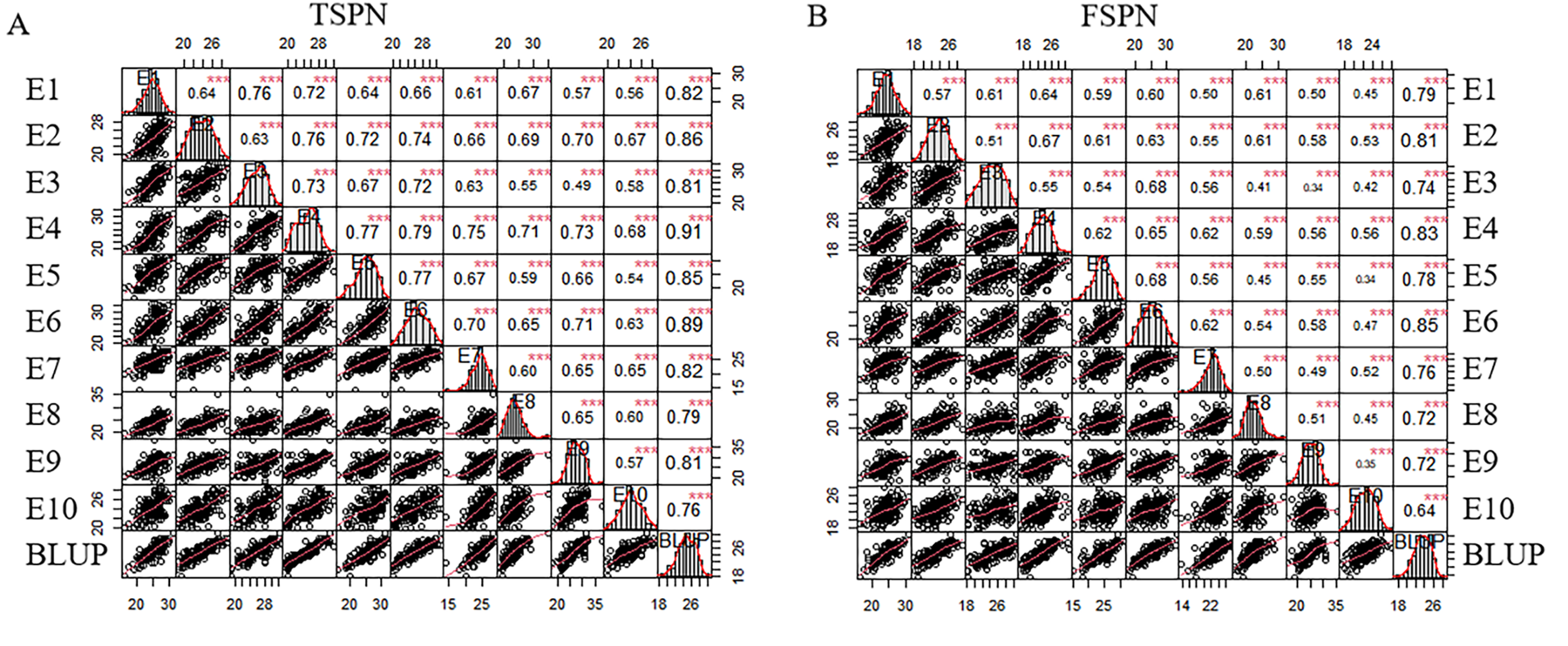

Supplement: Supplementary Figure 1 — Phenotypic performances, distribution, and correlation coefficients for total spikelet number per spike (TSPN) (A) and fertile spikelet number per spike(FSPN) (B) of parents and 10-A/B39 lines in ten environments and their corresponding best linear unbiased prediction (BLUP) values; *** represents significance at P < 0.001. [file Image_1.tif]

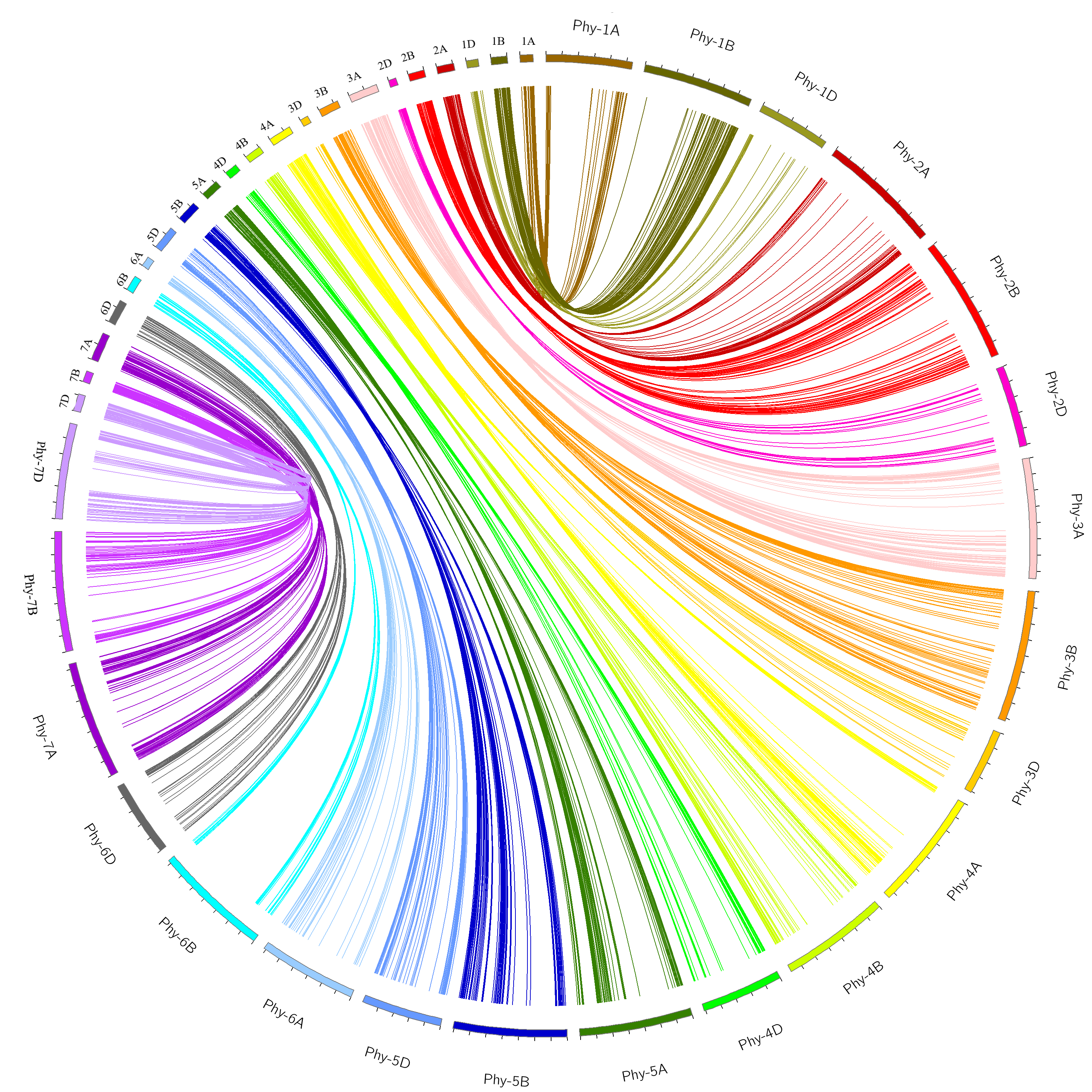

Supplement: Supplementary Figure 2 — Schematic representation of the syntenic relationships between a given marker in wheat genetic and physical maps. 1A to 7D represent the 21 wheat chromosomal genetic maps released in this paper; Phy-1A to Phy-7D represent the 21 wheat chromosomal physical maps. [file Image_2.tiff]
